# The Fever Tree: from Malaria to Neurological Diseases

**DOI:** 10.3390/toxins10120491

**Published:** 2018-11-23

**Authors:** Sara Eyal

**Affiliations:** Institute for Drug Research, School of Pharmacy, The Hebrew University of Jerusalem, Jerusalem 91120, Israel; sarae@ekmd.huji.ac.il

**Keywords:** quinine, quinidine, cinchona, malaria, atrial fibrillation, Brugada syndrome, prolonged QT syndrome, restless leg syndrome, Alzheimer’s disease, epilepsy

## Abstract

This article describes the discovery and use of the South American cinchona bark and its main therapeutic (and toxic) alkaloids, quinine and quinidine. Since the introduction of cinchona to Europe in the 17th century, it played a role in treating emperors and peasants and was central to colonialism and wars. Over those 400 years, the medical use of cinchona alkaloids has evolved from bark extracts to chemical synthesis and controlled clinical trials. At the present time, the use of quinine and quinidine has declined, to a large extent due to their toxicity. However, quinine is still being prescribed in resource-limited settings, in severe malaria, and in pregnant women, and quinidine made a limited comeback in the treatment of several cardiac and neurological syndromes. In addition, the article presents more recent studies which improved our understanding of cinchona alkaloids’ pharmacology. The knowledge gained through these studies will hopefully lead to a wider use of these drugs in precision medicine and to design of new generation, safer quinine and quinidine derivatives.

## 1. From Bark to Chemical Synthesis

In 1928, C.J.S. Thompson described in the British Medical Journal a “picturesque story” of the discovery of the cinchona bark qualities. The story, according to this paper, was about an Indian who became stricken with fever and drank the water of the lake into which a cinchona tree has fallen, fell asleep, and awoke to find that his fever was cured [1]. Whether the Peruvian natives knew about the therapeutic effects of cinchona or not, and whether malaria was known in the early modern world or not, remain a matter of debate [2]. The people of Peru were knowledgeable of medicinal plants and strongly adhered to their traditional customs. However, they made no medical use of cinchona, and in many cases would rather die than consume a remedy which they considered so dangerous [3,4].

The European story of the discovery of the tree involves the Spanish Countess of Cinchón, the wife of the Viceroy of Peru, who, in the 1630s, was ill with intermittent fever in the palace of Lima. When the news on her fever reached Don Francisco Lopez de Canizares, the Corregidor of Loxa (who had himself been cured of fever by means of the same drug), he provided a packet of the powdered bark to the countess’s physician, Dr. Juan de Vega. Upon treatment, the countess rapidly recovered and ordered the preparation and distribution of a large amount of the powder to those suffering from fever, and brought it back with her when she returned to Europe, in 1640. The preparation thus became known as the “countess’s powder” [1]. However, the Peruvian bark probably arrived to Europe earlier, in the 1630s, by Spanish Jesuit missionaries, who were its strongest promoters [2,5,6].

Although today malaria is considered a disease of tropical regions, in the 17th century it was common in many parts of Europe, killing peasants, kings and popes. The term Malaria itself was Italian, describing “bad air” (mal’ aria, or male d’aria) that was believed to cause certain types of fever [7]. No effective cure was found, and patients were mostly treated by bleeding. Yet, despite therapeutic successes and support from the Vatican, cinchona was not introduced into general use. The delay of acceptance resulted from its lack of efficacy in curing fevers other than those of malaria, the use of inferior quality barks sold by some dealers, inexperience in the use of the bark, and the conflict with Galenic medicine. In Protestant England, the support of the Vatican worked against the acceptance of the bark due to fears of popish plots [1,5,8].

It was the English apothecarian Robert Talbor who provoked public awareness of cinchona, in the late 17th century. Robert Talbor exploited the fears of the public and used cinchona as a secret remedy, not disclosing that the central ingredient was the Peruvian bark. He successfully treated the King of England Charles II and the young son of Louis XIV using the mysterious powder [1,8]. Louis XIV bought the recipe of the powder, under the condition that it is not read until after Talbor’s death. Talbor’s secret turned out to be the questionable cinchona [2,5]. Talbor used several preparations of the bark, including strong infusions and a tincture [1]. In 1677 the Peruvian bark first appeared officially in the London Pharmacopoeia as “Cortex Peruanus” [1,2].

In 1735, the Academy of Science of Paris decided to send a delegation that included the botanist Joseph de Jussieu to South America to define the curve and shape of the earth and to study the plants of South America. Joseph de Jussieu named the Cinchona tree in French “l’arbre des Fièvres” (the Fever Tree) [8]. He wrote: “They call it *yara chucchu cara chucchu*. *Yara* means tree, *cara* the bark, *chucchu* cold of the fever, so to say the tree of intermittent fever” [2]. Unfortunately, de Jussieu’s report remained unpublished until 1936 [9]. In 1742 the botanist Carl Linnaeus established the genus Cinchona, and called the tree in honor of the Countess of Cinchón [1].

**Figure 1:**
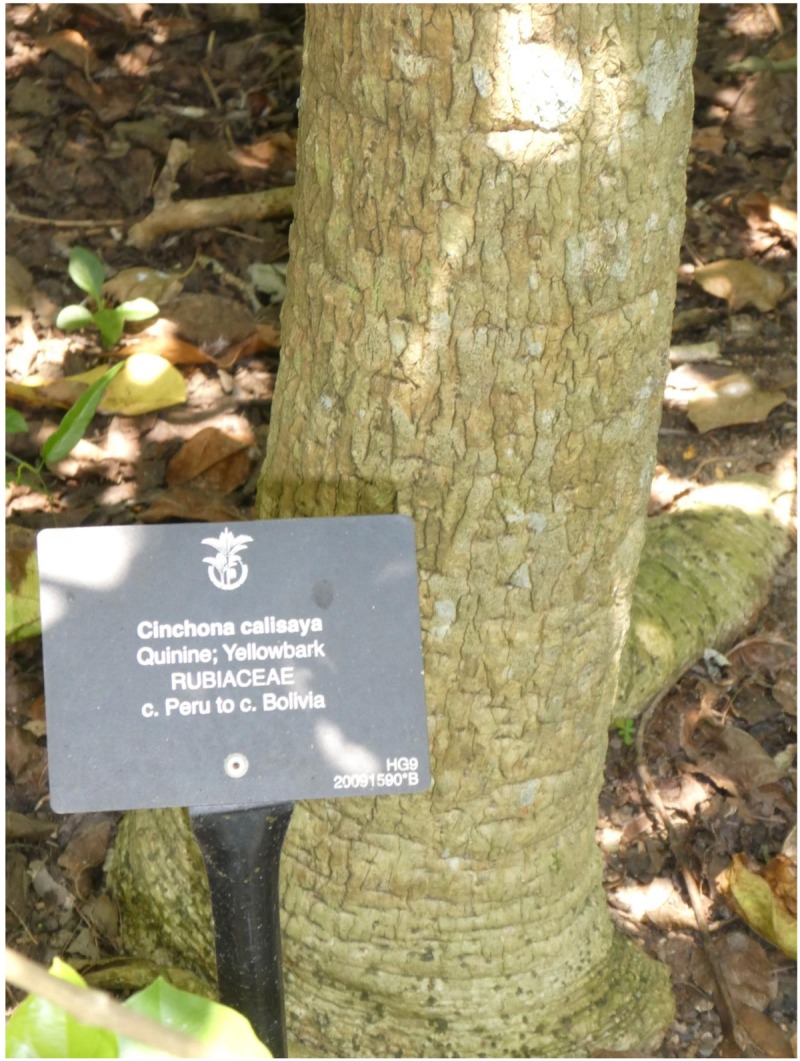
*Cinchona calisaya*. 2018. Photo by the author, Singapore Botanic Gardens.

Transport of the bark to Europe began in the mid-17th century, and continued until the 19th century. By the mid-19th century Europeans began claiming that the non-sustainable manner in which the natives of Peru collected the bark threatened the trees. The possibility that there would soon be a shortage of the drug, combined with the colonizing efforts of Europeans, prompted attempts to establish cinchona tree plantations in tropical zones. Meanwhile, Peru and surrounding countries had started to outlaw the export of cinchona seeds to maintain their monopoly on the bark. In the 1860s, the English trader Charles Ledger and his native servant Manuel Icamanahí spent four years in the Yungas, Bolivia, collecting cinchona seeds. On one of this trips, Icamanahí was arrested, imprisoned, and beaten to death. The seeds of *Cinchona calisaya* (Figure 1) he had collected did get sent to London. However, the seeds were rejected by the director of Kew Gardens and have instead become the basis for the Dutch development of the Cinchona plantations of Java, which, before World War I, provided 85% to 90% of the world consumption of quinine [1,2,5,6].

Until the early 1800s, the cinchona bark was still used in its crude state. In 1810 Bernardino Antonio Gomez isolated from the bark the alkaloid which he called “cinchonino.” In 1820, the French chemists Pierre-Joseph Pelletier and Joseph Caventou proved that “cinchonino” was a mixture of two distinct alkaloids, which they termed “quinine” and “cinchonine.” The next advance was made in 1833 by Henry and Delondre, who isolated quinidine. In 1844 cinchonidine was discovered by Winckler and in 1853 it was named quinidine by Pasteur [1,7,8].

**Figure 2:**
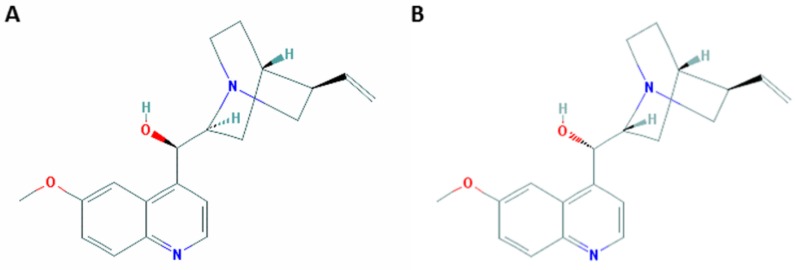
The molecular structures of quinine (**A**) and quinidine (**B**) (from PubChem; CID 3034034 and CID 441074, respectively).

The complexity of cinchona alkaloids made their chemical synthesis challenging. Quinine and quinidine have heterocyclic structures, with four chiral centers and thus 16 stereoisomers (Figure 2). In 1918, Paul Rabe and Karl Kindler published a method for the final steps in the synthesis of quinine (and quinidine) [10], but their work has become the subject of a 90 year-dispute [11]. In the mid-1940s, Robert Woodward and William Doering reported on a 17-step ‘total synthesis’ of quinine to yield *d*-quinotoxine [12,13], from which only three final steps were required to obtain quinine, according to Rabe’s work [14]. Woodward’s and Doering’s synthesis was disputed as well, especially by Gilbert Stork, who was in 2001 the first to report on stereoselective total synthesis of quinine [11,15]. In 2007, Robert Williams repeated Rabe’s reaction under 1944 conditions and obtained quinine, thereby demonstrating that the total synthesis of quinine by Woodward-Doering and Rabe-Kindler was indeed possible [16].

## 2. The Rise and Descent of Cinchona Alkaloids as Antimalarial Drugs

The earliest large scale observational study on the use of cinchona alkaloids in the treatment of malaria was conducted in 1866 to 1868 in Southern India by the Madras Chinchona Commission. The report of the commission described 2472 cases of paroxysmal malarial fever, among which 846 were treated with quinine, 664 with quinidine, 559 with cinchonine and 403 with cinchonidine. A total of 2445 patients were cured and 27 failed. The respective ratios of failure per 1000 cases were 7, 6, 23, and 10 [17,18]. According to a report of the Madras Commission on 846 patients, the toxic effects of quinidine were similar to those of quinine [18]. Despite the equivalent antimalarial efficacy and similar toxicity of quinine and quinidine, after 1890 quinine has been the most commonly used alkaloid, predominantly due to the supply of bark from Javan cinchona, which contained a higher proportion of quinine [19].

During World War 1, quinine was the only antimalarial drug available. The allied armies obtained their quinine supply from cinchona plantations in the Asian Dutch colonies, whereas naval blockade led to shortages of quinine among the German and the Austro-Hungarian forces [20]. The prolonged treatment durations (weeks), various experimental routs of administration, and toxic effects of quinine, led British medical officers to assume that soldiers would not be compliant. In Salonika (Greece), where malaria causalities were particularly high, soldiers had to be observed by officers ingesting their daily quinine dose with water. Ronald Ross, consultant Physician to the Mediterranean Expeditionary Force who discovered the mosquito transmission of malaria, created quinine concentration camps in England and France for intensive enforced drug treatment. Under those treatment protocols, blood-stage parasites could be eliminated, yet the drug did not affect the hepatic forms of the parasite (largely *Plasmodium vivax*) and efficacy of the treatment was not clear [20,21]. In 1917, Sir William Osler commented that “the man who could not treat malaria successfully with quinine should give up the practice of medicine” [21]. Quinine was the main treatment of malaria until the 1920s, when chloroquine was introduced. However, with the development of resistance to chloroquine, quinine has again become an optional treatment.

**Figure 3:**
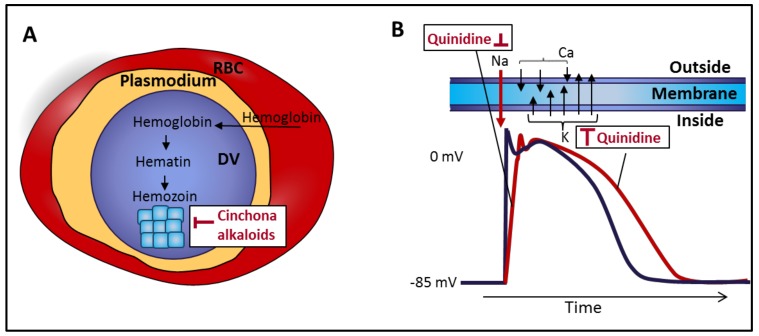
The molecular mechanisms of the antimalarial and antiarrhythmic activities of cinchona alkaloids. (**A**) Antimalarial activity of cinchona alkaloids. Hemoglobin is digested in digestive vacuoles (DV) of the intraerythrocytic forms of plasmodium parasites to hematin, which is toxic to the parasite. The parasite converts hematin into hemozoin thus reducing its detrimental effects. Cinchona alkaloids inhibit the growth of hemozoin crystals, thus interfering with heme detoxification. RBC, red blood cell. (**B**) Antiarrhythmic activity of quinidine. Quinidine reduces sodium and potassium currents and prolongs the duration of action potentials and the QT interval of the electrocardiogram (ECG).

Quinine acts against the asexual erythrocytic forms of the four species of human malaria parasites. It is not active against liver stage parasites [22]. Ronald Ross’s discovery of Anopheles mosquitos as the malaria vector was based on his identification in the stomach wall of Anopheles mosquitos bodies which “contained a few granules of black pigment absolutely identical in appearance with the well known and characteristic pigment of the parasite of malaria” [23,24]. This pigment was shown to be an insoluble crystalline material called hemozoin, whose growth was inhibited by quinoline-containing drugs via inhibition of a heme polymerase enzyme [25] (Figure 3A). Recently, 120 years after Ross’s discovery, Olafson et al. demonstrated that the adsorption of quinine and other quinolone drug to the crystal surface is the main mechanism of crystal growth inhibition [26].

## 3. Introduction of Cinchona Alkaloids as Antiarrhythmic Agents

The effect of quinidine on cardiac function was first reported in 1914 by Wenckebach [27], who was aware of quinine use for more than 60 years before his studies. Wenckebach wrote “Among the older generation of clinicians there was a good number, and I knew some of them, who nearly always gave digitalis in combination with quinin. On being asked why they did it, they could not give me an exact reason for it; but they found that digitalis was better supported with quinin and that they could give larger doses without getting disagreeable symptoms.” Wenkebach was familiar with quinine’s toxicity and had limited success in his patients with oral quinine in the treatment of atrial fibrillation. However, he noted that quinine worked best in cases when the onset of the arrhythmia was recent and “in those cases in which there is not much wrong with the heart”—observations that still stand [28]. Wenkebach’ colleague, Winterberg, observed that intravenous administration was often more effective than the oral form [27]. Walter Frey, in 1918, conducted the first systematic study on the effect of cinchona alkaloids in patients with atrial fibrillation and found that quinidine was more effective than quinine in cardioverting to sinus rhythm [29]. This was later confirmed by others, particularly Sir Thomas Lewis who demonstrated that quinidine sulfate prolonged the refractory period of cardiac tissue [30]. Lewis, however warned against the uncontrolled use of quinidine [30]. Despite the potential toxicity, the use of quinidine had become common for the next 40 years [28]. In 1964, Selzer and Wray described attacks of syncope (“Quinidine Syncope”), in most cases apparently related to paroxysms of ventricular flutter or fibrillation, in patients receiving standard doses quinidine [31]. All patients were concurrently treated with digitalis, which was suggested as a contributing factor but not shown to be implicated in the adverse reaction. The concerns about ventricular fibrillation led to a dramatic decrease in the use of quinidine for the treatment of atrial fibrillation in favor of newer antiarrhythmic medications [32]. However, increased mortality in patients treated with the newer antiarrhythmic drugs encainide or flecainide in the Cardiac Arrhythmia Suppression Trial [33] boosted the use of quinine after cardioversion of atrial fibrillation [34].

Quinidine is a class IA antiarrhythmic which primarily blocks sodium channels but is also a broad-spectrum potassium channel blocker (Figure 3B). As a consequence, quinidine prolongs the cardiac action potential duration and prolongs the QT interval of the electrocardiogram (ECG). Antagonism of α adrenergic receptors and vagal inhibition may be associated with marked hypotension and sinus tachycardia when quinidine is given intravenously. The vagolytic effect of quinidine can result in increased transmission of atrial tachycardias via the atrioventricular (AV) node [35].

## 4. Toxicity of Quinine and Quinidine Still Limits Their Use

Quinine has been used as a medicine before 1938, when the US Food, Drug, and Cosmetic Act was enacted. Therefore, it was not initially regulated by the US Food and Drug Administration (FDA). Quinine has a low therapeutic index, with a total oral daily dose for adults of about 2–8 g. The target plasma concentration of unbound quinine for treating malaria is roughly 0.8 µg/mL (corresponding to approximately total concentrations of 8 µg/mL) [36]. At full therapeutic or excessive doses, quinine is associated with a triad of dose-dependent toxicities: Cinchonism, hypoglycemia, and hypotension. Mild forms of cinchonism include tinnitus, slight impairment of hearing, headache, nausea, visual disturbances, and postural hypotension. These symptoms occur at free concentrations above 2 µg/mL and disappear soon after the drug is withdrawn [22]. Prolonged medication or large doses may lead to more severe symptoms, including vertigo, vomiting, abdominal pain, diarrhea, marked auditory loss, diminished visual acuity or loss of vision, and cutaneous manifestations (flushing, sweating, rash, angioedema). Hypoglycemia (and hyperinsulinemia) is also common and can be severe. Hypotension may occur if the drug is given intravenously too rapidly. At therapeutic plasma concentrations, cardiac complications are rare, although mild QTc prolongation may be observed. Acute over-dosage may cause life-threatening cardiac dysrhythmias [19,22,37]. In 2004, quinine derivatives were included in the list of “Medications Which can Kill a Toddler with One Tablet or Teaspoonful” [38].

Quinine can additionally cause severe immune-mediated systemic reactions [39]. A systematic review of adverse reactions to quinine identified 142 patients with definite or probable evidence for a causal association of quinine with acute, immune-mediated reactions. These reactions included mostly hematological adverse effects such as disseminated intravascular coagulation, hemolytic anemia and thrombocytopenia [40]. A rare hypersensitivity reaction to quinidine is “Blackwater fever”—the triad of massive hemolysis, hemoglobinemia, and hemoglobinuria which lead to renal failure and may be fatal [22]. Quinine can additionally cause milder hemolysis, especially in patients with glucose-6-phosphate dehydrogenase deficiency [22]. Other immune-mediated reactions which have been associated with quinine involve the skin, the liver and the kidneys [40].

The most common adverse effect during quinidine therapy is diarrhea, usually occurring within the first several days of quinidine therapy. Diarrhea-induced hypokalemia may potentiate the arrhythmia torsades de pointes due to quinidine. Similar to quinine, quinidine therapy has been associated with immunological reactions, including thrombocytopenia, hepatitis, bone marrow depression, and lupus syndrome. These effects may occur at therapeutic plasma quinidine concentrations. At elevated plasma levels, quinidine also can produce cinchonism, which can be managed by dose reduction [35].

Quinidine is more cardiotoxic than quinine. Marked QT interval prolongation and torsades de pointes may occur in 2–8% of patients treated with quinidine. High plasma concentrations of quinidine achieved by very high doses formerly used to try to convert atrial fibrillation to normal rhythm have been associated with marked sodium channel blockade and increased risk of ventricular tachycardia. This dosing approach is not being used today, and quinidine-induced ventricular tachycardia is unusual [35]. Quinidine can exacerbate heart failure, but is well tolerated in most patients with congestive heart failure, possibly because of its vasodilating actions [35].

## 5. Current Uses of Quinine and Quinidine

Today, the treatment of choice for patients with severe malaria is artesunate. However, quinine is used in resource-limited settings and in the treatment of pregnant women [6,19,41]. According to the US Centers for Disease Control and Prevention (CDC), quinine sulfate, combined with doxycycline, tetracycline, or clindamycin is a second-line treatment option for *Plasmodium falciparum* infections acquired in areas with chloroquine resistance. The treatment is given for seven days for infections acquired in Southeast Asia and for three days for infections acquired in Africa or South America. The combination is aimed to enhance quinine efficacy, shorten the duration of therapy and limit toxicity [22,42]. Persons acquiring chloroquine-resistant *P. vivax* infections (mainly in Papua New Guinea or Indonesia) may be treated with quinine sulfate plus doxycycline or tetracycline. A combination of quinine sulfate and clindamycin is recommended for pregnant women diagnosed with uncomplicated malaria caused by chloroquine-resistant *P. falciparum* infection [41]. Similar recommendations for the treatment of uncomplicated malaria have been formulated by the World Health Organization (WHO) [43]. However, in most of Africa, quinine is used as monotherapy, likely due to the higher costs of the combined therapy [19]. More controlled studies conducted since the study by the Madras Commission demonstrated that quinidine is more potent as an antimalarial and more toxic than quinine [22]. Since 1991, quinidine gluconate has been the only parenterally administered antimalarial drug available in the United States, and is being used in the treatment of severe malaria [42].

Quinine has been prescribed for idiopathic muscular cramps or restless leg syndrome [44], because it increases the tension response to a single maximal stimulus delivered to muscle and the refractory period of muscle [22]. By 2012, reported off-label treatment of these conditions consisted approximately 92% of quinine use in the United States [45]. In 2010, a subcommittee of the American Academy of Neurology concluded that the use of this drug to treat muscle cramps should be avoided [45]. This conclusion was supported by a recent study that found that long-term quinine exposure was associated with increased mortality in a dose-dependent manner [44].

**Figure 4:**
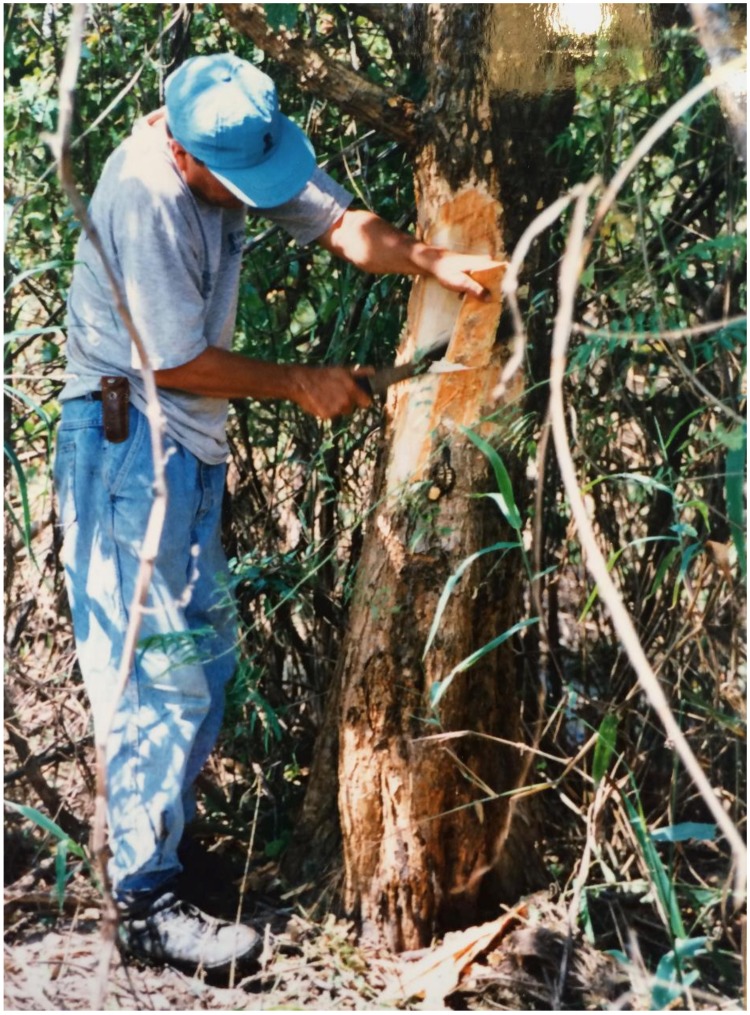
Harvesting the bark of an identified tree for medical purposes. The bark was suspended in boiling water for several hours and the extract was applied externally overnight for treating a skin infection in the author’s foot. 1995. Photo by the author, Llanos de Moxos, Bolivia.

In 2005, the FDA approved quinine for the treatment of uncomplicated *P. falciparum* malaria and in 2006 it removed over-the-counter quinidine products for nocturnal leg cramps from the market [45]. The labeling was updated in 2010 to include a Boxed Warning against the use of quinine for the treatment or prevention of nocturnal leg cramps [37] and a Risk Evaluation and Mitigation Strategy (REMS) was approved. This REMS included a Medication Guide and a communication plan to prescribers and to professional medical societies, informing them about the risk and the appropriate use of quinine [45]. Quinine is currently approved by the FDA for the treatment of malaria only [46]. However, in some countries, including Canada, tablets can be purchased without a prescription, and quinine-containing beverages are available everywhere [39,40]. Quinine may be also found in cosmetics, e.g., shampoos [47]. The majority of medical quinine use in the United States continues to be associated with the off-label indications. In a review of the FDA’s adverse event reporting system database, in only 1 of 38 cases of serious adverse reactions reported to the agency between 1 April 2005 and 1 October 2008, quinine was used for the treatment of malaria; in the other cases, it was indicated for muscle cramps, restless leg syndrome, or neuropathy [45]. In traditional medicine, the barks of cinchona and other trees are still being used for treating a variety of disease conditions (Figure 4).

Atrial fibrillation is currently being largely treated by agents more effective or safer than quinidine. Hence, quinidine’s overall use as an antiarrhythmic drug has declined substantially [32]. However, this drug has made a comeback in the prevention of arrhythmias in patients with short QT syndrome or Brugada syndrome [48]. Short QT syndrome is a rare genetic disease characterized by the combination of a short QT interval and life-threatening arrhythmias [49]. Quinidine is an effective alternative because it prolongs the QT interval. It might also prevent ventricular fibrillation [48]. Brugada syndrome is an inherited disease characterized by ventricular arrhythmias and increased risk of sudden death. In patients with Brugada syndrome, quinidine is used for treating electrical storms or frequent shocks delivered by implantable cardioverter-defibrillators (ICD) or as an alternative for patients with contraindications for ICD implantation [48,50,51]. Quinidine’s elimination half-life (3–19 h) is shorter than that of quinine (11 h; up to 18 h in patients with malaria). Extended-release formulations can potentially improve compliance to quinidine [42,52].

A fixed-dose preparation of quinidine sulphate and dextromethorphan hydrobromide (Nuedexta) is the first treatment approved in the United States and in Europe for pseudobulbar affect, a neurological disorder characterized by uncontrollable episodes of exaggerated emotional expression that occurs in adults with neurological damage conditions [53]. In this combination, low-dose quinidine is utilized to inhibit the metabolism of dextromethorphan by cytochrome P450 (CYP) 2D6, thus increasing the plasma (and brain) concentrations of dextromethorphan. Dextromethorphan is an agonist of CR-1 receptors in brain regions associated with emotions [54].

In 2015, a phase II randomized controlled study demonstrated that patients with Alzheimer’s disease who received the quinidine/dextromethorphan combination had lower occurrence and severity of agitation than patients who received a placebo [55]. The combination was not associated with cognitive impairment, sedation or clinically significant QT prolongation. Potentially relevant mechanistic actions of the combination include inhibition of serotonin and norepinephrine reuptake and antagonism of *N*-methyl-d-aspartate and nicotinic α3β4 receptors [53].

Recently, quinidine has been evaluated as a precision therapy for people with seizures associated with mutations of the potassium channel KCNT1. *KCNT1* mutations were discovered in autosomal dominant nocturnal frontal lobe epilepsy (ADNFLE) [56] and in malignant migrating focal seizures of infancy [57]. *KCNT1* mutations cause a gain of function in the potassium channel, and in vitro studies in Xenopus oocytes demonstrated that quinidine reverses the increased channel function in a concentration-dependent manner [58]. Several case reports described the repurposing of quinidine for treating epileptic encephalopathy due to *KCNT1* mutations, although therapeutic effectiveness was inconsistent [59,60,61,62]. Yet, the *KCNT1* gain-of-function appeared to provide a clear target for quinidine. This has motivated a small crossover, add-on trial in which quinidine was given to six adults with ADNFLE carrying *KCNT1* mutations [63]. Unfortunately, this study failed to show efficacy prolonged QT interval in two patients limited the dose given to the others, and increased plasma concentrations of digoxin during concurrent treatment with quinidine was first reported in 1978, in 25 of 27 patients [64]. The interaction resulted in reversible gastrointestinal adverse events in 16 patients, ventricular premature depolarizations in three patients and sudden death of one patient. Inhibition of the major efflux transporter P-glycoprotein (P-gp), the major route of digoxin elimination, has been later implicated in the quinidine-digoxin interaction [65]. This interaction seems now to explain the “Quinidine Syncope” observed by Selzer and Wray in the 1960s. Current practice requires reduction of digoxin dosage when combined with quinidine [35]. Quinidine has become an established P-gp inhibitor suggested by the FDA for preclinical and clinical studies [66] and is particularly used in studies assessing P-gp’s role at the blood-brain barrier (BBB) [67]. Recently, quinidine was used as the P-gp inhibitor in a positron emission tomography study that evaluated P-gp modulation at the human BBB [68]. Quinidine was selected based on its ability to inhibit P-gp in vitro (effective inhibitory concentration–EC_50_: 0.9 µM) and its unbound therapeutic plasma concentration–Cu (1.3 µM; Cu/EC_50_: 1.4), which predicted effective P-gp inhibition at the human BBB. In that study, quinidine did not completely inhibit BBB P-gp. However, the authors suggested that it has the potential to produce clinically significant CNS drug interactions with other P-gp substrate drugs that have a narrow therapeutic window.

## 6. Conclusions and Prospective

The history of cinchona and its alkaloids provides examples of the role of politics, religion and war in the introduction of medications into the clinic. Cinchona alkaloids, likely recognized hundreds of years ago by the natives of Peru as a toxin, are still being used, both for approved indications and off-label. Besides therapeutic successes, the use of cinchona alkaloids has been associated with life-threatening adverse reactions. Improved labeling and education of prescribers and the public, together with better understanding of the pharmacology of cinchona alkaloids, may minimize such adverse reactions [40]. At the same time, the knowledge gained on the molecular mechanisms and pharmacokinetics of these compounds may support their use in precision medicine, by addressing the deficits from genetic mutations. However, the study of quinidine use for the treatment of epilepsy should serve as a reminder that even a drug that has been on the market for decades cannot be repurposed without adequate trials of its efficacy and safety. It is hoped that the development of better and safer derivatives of cinchona alkaloids will extend their therapeutic use.
